# Development of a novel light-up probe for detection of G-quadruplexes in stress granules

**DOI:** 10.1038/s41598-022-17230-y

**Published:** 2022-07-28

**Authors:** Keisuke Iida, Natsumi Suzuki, Ayano Sasaki, Shunsuke Ishida, Takayoshi Arai

**Affiliations:** Soft Molecular Activation Research Center (SMARC), Chiba Iodine Resource Innovation Center (CIRIC), Molecular Chirality Research Center (MCRC), and Department of Chemistry, Graduate School of Science, Chiba University, 1-33 Yayoi, Inage, Chiba , 263-8522 Japan

**Keywords:** DNA, RNA, Nucleic acids, Small molecules

## Abstract

G-quadruplexes (G4s) regulate various biological processes in cells. However, cellular imaging of dynamically forming G4s in biomolecular condensates using small molecules has been poorly investigated. Herein, we present a fluorescent light-up probe with the ability to selectively stabilize G4s and enhance fluorescence upon G4 binding. The foci of the probe were mainly observed in the nucleoli. These were co-localized with anti-fibrillarin antibodies and anti-G4 antibodies (BG4). Moreover, we tested detection of G4 in stress granules using the developed probe. Stress granules were induced through treatment with not only thapsigargin, but also known G4 ligands (pyridostatin, RHPS4, and BRACO-19). In the stress granules, co-localization between the probe, BG4, and stress granule markers (TIA1 and G3BP1) was detected. We present a practical light-up probe for G4s in stress granules, providing potential targets for G4 ligands.

## Introduction

G-quadruplexes (G4s) are higher-order structures of nucleic acids formed from Hoogsteen base pairs in guanine-rich sequences. DNA G4s are especially concentrated in important regions such as telomeres^1^, promoters^2^, and CpG islands^3^, and genome-wide high-throughput G4 sequencing has identified more than 700,000 DNA G4 sites in the human genome^4^. The G4-sequencing approach has also revealed the existence of RNA G4s in more than 3000 mRNA strands^5^. Moreover, G4s regulate a wide variety of biological processes, including transcription, translation, replication, epigenetic reprogramming, and stress granule (SG) formation^6–8^. In recent years, the observation of the dynamic formation of G4s in cells has been reported using an anti-G4 antibody, and the significance of G4s has grown in biology^9–11^. In the past decades, a wide variety of G4 ligands have been developed^12–14^ to investigate anti-cancer effects through telomerase inhibition and/or transcriptional repression, e.g., acridine compounds (BRACO-19^15^ and RHPS4^16^), macrocyclic compounds^17^, naphthalene diimide^18^, pyridostatin^19^, CX-3543^20^ and DOTASQ^21^. More recently, fluorescence G4 ligands^22–28^ as represented by CyT^29^, SQgI^30^, DAOTA^31^, QUMA-1^32^, and TASQ^33^, have been reported for the detection of G4s in vitro and in cells. Moreover, imaging of cellular G4s using light-up ligands as markers remains a challenging task; in particular, visualization of G4s in SGs has only been reported using an anti-G4 antibody^34^. The association between G4s and SGs has received great attention in chemistry and biology because nucleic acids and proteins drive the generation of various biomolecular condensates through liquid–liquid phase separation^35^. In this study, we developed novel light-up G4 ligand **1** and demonstrated that it gives cellular signals when co-localized with anti-G4 antibodies and SG markers. SGs were induced using the well-known endoplasmic reticulum (ER) stress inducer thapsigargin, and further investigation revealed that G4 ligands can also be utilized as exogenous stimuli for SG assembly.

## Results

**Figure 1:**
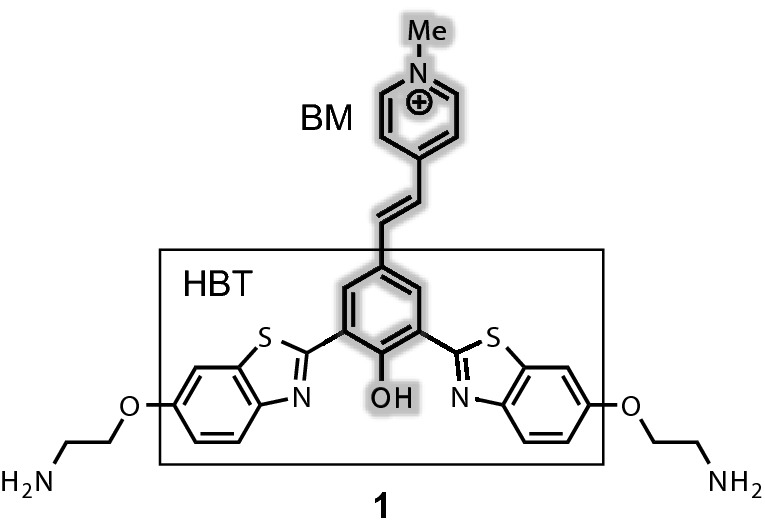
Structure of light-up G4 ligand **1**.

We designed a fused skeleton of Brooker's merocyanine (BM) and 2-hydroxybenzothiazole (HBT) as a fluorescent G4 ligand (Fig. 1). BM is known as a solvatochromic dye, and its fluorescence is affected by the solvent^36–39^. Moreover, HBT is well known as an excited-state intramolecular proton transfer (ESIPT) dye, and is also sensitive to changes in the surrounding environment^40–43^. Therefore, the fluorescence properties (peak wavelength and fluorescence intensity) of the large aromatic skeleton, featuring BM and HBT moieties, are expected to change upon G4 binding. Additionally, cationic amino side chains were introduced to the core skeleton, providing water solubility and enabling electrostatic interactions with phosphate backbones (Fig. 1). The synthesis of **1** was readily achieved in several steps from 2-iodoisophthalic acid (see Supplementary information).

To investigate the G4 binding ability, **1** was evaluated using a fluorescence resonance energy transfer (FRET) melting assay^44,45^ with fluorescence-labeled known DNA (F-telo-T, F-myc-T, F-kit-T, and F-thr-T) and RNA (F-VEGF-T, F-TRF2-T, and F-TERRA-T) G4-forming sequences. The *T*_1/2_ values of **1** at various concentrations, which corresponded to 2.5–10 equivalents, are summarized in Fig. S1. In all cases, the *T*_1/2_ values increased, and some showed increases of more than 20 °C, whereas no significant changes in the *T*_1/2_ values were observed when using a stem loop sequence (F-ds26-T) as a non-G4-forming sequence. This indicates that **1** stabilizes DNA and RNA G4s selectively.

**Figure 2:**
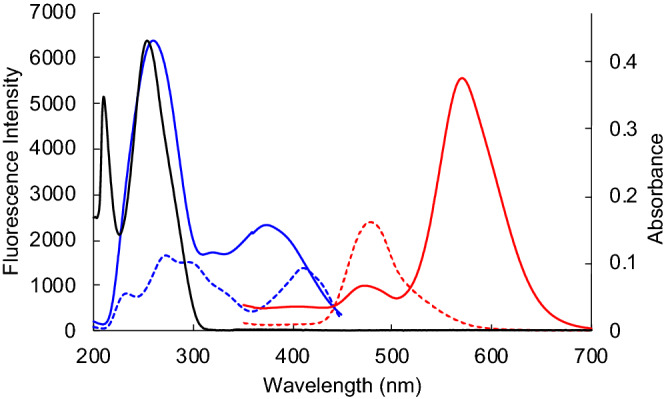
Excitation (blue dashed line) and fluorescence (red dashed line) spectra of **1** (1 µM) in water, excitation (blue solid line) and fluorescence (red solid line) spectra of **1** (1 µM) in the presence of 1 equiv of c-kit G4 DNA, and ultraviolet–visible (UV–vis) spectrum of c-kit G4 DNA (2 µM).

**Figure 3:**
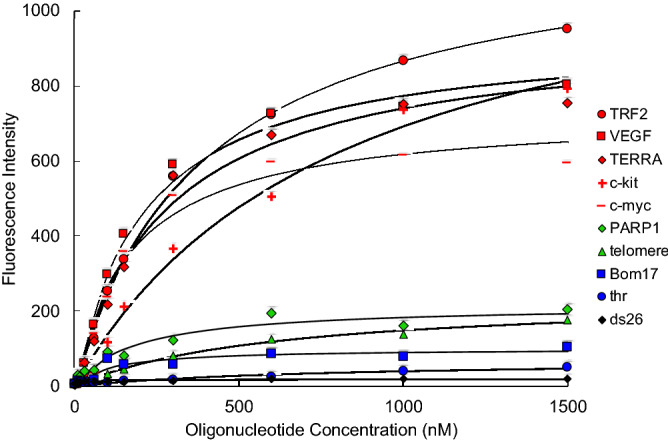
Results of fluorescence titration of **1** (50 nM) in the presence of various G4s and non-G4s. Fluorescence intensities of **1** at 575 nm (ex. 260 nm) were plotted against various nucleic acid concentrations. The solid lines are the fitted curves assuming 1:1 stoichiometry.

The biophysical properties of **1** were investigated using fluorescence spectroscopy. The absorption and fluorescence emission spectra of **1** revealed negative solvatochromism in organic solvents (Fig. S2), and the fluorescence of **1** suppressed in water, which is probably ascribed to aggregation-caused quenching (ACQ)^46^ and inhibition of ESIPT^42^ (see Supplementary information, Fig. S3 and S4). On the basis of these results, we next measured the fluorescence spectrum of **1** with the addition of a G4 nucleic acid (c-kit G4; a G4-forming sequence that mimics the c-kit promoter) in an aqueous buffered solution. The signal intensity at 575 nm clearly increased in the presence of c-kit G4s (Fig. 2). Titration experiments at 575 nm were performed using various G4-forming sequences and dsDNA, similar to the FRET melting study (Fig. S1), and the fluorescence intensities are shown in Fig. 3. The addition of G4s enhanced the fluorescence of **1** in a dose-dependent manner. In particular, **1** showed high fluorescence enhancement in the presence of a wide range of parallel G4s (red symbols; TRF2, VEGF, TERRA, c-kit, and c-myc), and modest light-up was observed in the case of hybrid (green symbol; telomere and PARP1) and anti-parallel (blue symbol; thr and Bom17) G4s. However, no significant changes were observed for dsDNA. The fluorescence enhancement of **1** is likely caused by the recovery of ESIPT along with stacking to form a G-quartet because a G-quartet is a hydrophobic environment. Moreover, the signal intensity near 260 nm in the excitation spectrum of **1** increased. These results indicated that **1** possessed light-up properties, that is ideal properties for the selective detection of G4s in cells.

**Figure 4:**
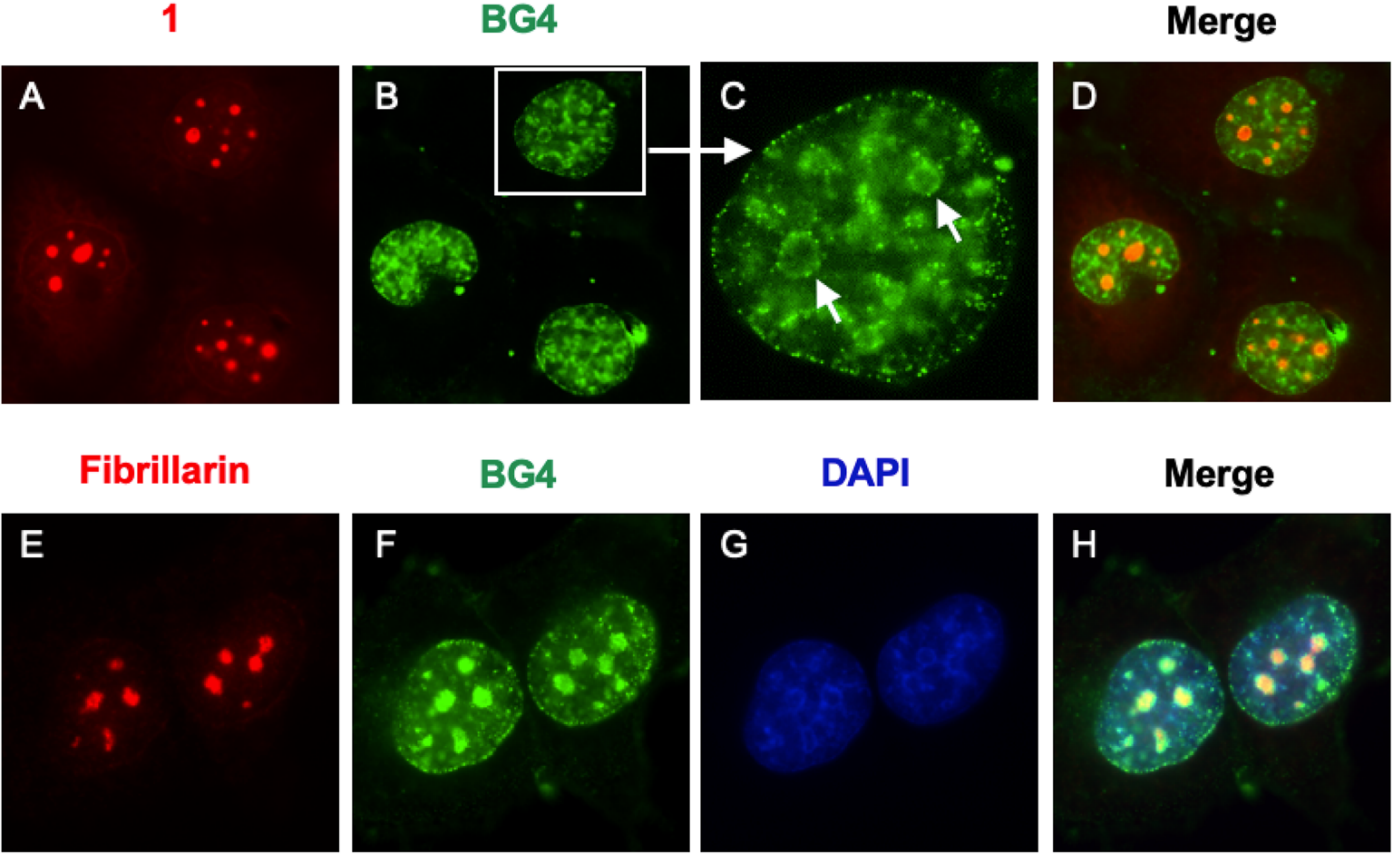
Cellular images of fixed H1299 cells. Co-staining for **1** and BG4: (**A**) **1**, (**B**) BG4, (**C**) magnified image for BG4, and (**D**) merged image. Co-staining for anti-fibrillarin antibody, BG4, and 4ʹ,6-diamidino-2-phenylindole (DAPI): (**E**) anti-fibrillarin antibody, (**F**) BG4, (**G**) DAPI, and (**H**) merged image. Fluorescence was analyzed using the following detection channels: “compound 1 channel” (ex. = 360/40 nm, em. = 605/70 nm), “BG4 channel” (ex. = 470/40 nm, em. = 525/50 nm), “Fibrillarin channel” (ex. = 560/40 nm, em. = 630/75 nm), and “DAPI channel” (ex. = 360/40 nm, em. = 460/50 nm).

After confirming the light-up properties of **1** in vitro, we examined the visualization of cellular G4s through immunocytochemistry. Fixed and permeabilized H1299 cells were treated with **1**, and the treated cells were co-stained with anti-fibrillarin antibodies as nucleolus markers (Fig. S5). As a result, the foci of **1** and fibrillarin were co-localized in the nucleoli. Subsequently, RNase and DNase digestion were carried out. RNase treatment clearly reduced the signal intensity of nucleolus foci, while DNase treatment had no effect, and only a decrease in the nuclear signal intensity was observed (Fig. S6). Furthermore, to investigate whether the nucleolus foci of **1** were folded G4 structures, the cells were treated with BG4 antibody as a G4 marker. The BG4 foci were found throughout the nucleus, including the nucleoli; however, in the case of co-staining with BG4 and **1**, the BG4 foci were partially displaced by **1** in the nucleoli (Fig. 4). This indicated that **1** mainly visualized RNA G4s in nucleoli, probably owing to the total amount of rRNA in the cells.

**Figure 5:**
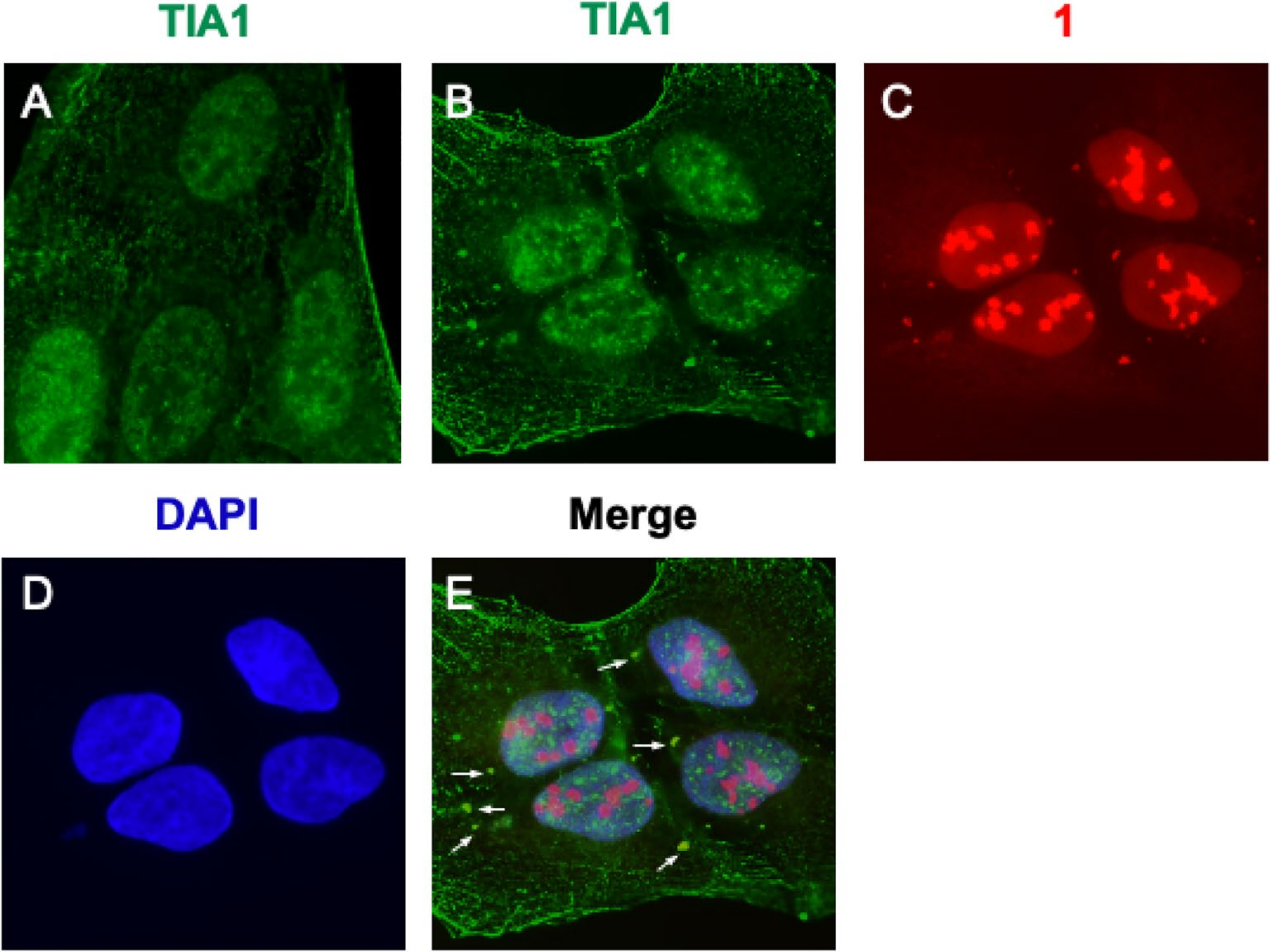
(**A**) Localization of anti-TIA1 antibody in untreated U2OS cells. (**B**–**E**) Co-localization of anti-TIA1 antibody and **1** in thapsigargin-stimulated U2OS cells. Fluorescence was analyzed using the following detection channels: “TIA1 channel” (ex. = 470/40 nm, em. = 525/50 nm), “compound **1** channel” (ex. = 360/40 nm, em. = 605/70 nm), and “DAPI channel” (ex. = 360/40 nm, em. = 460/50 nm).

To examine the practicality of **1** for the detection of cellular G4s as a marker, we visualized G4s in SGs. Previous reports have stated that a certain type of stress-induced RNA (tiRNA) triggers the assembly of SGs, which form as a result of G4s on tiRNA^47^. On the basis of reported conditions^48^, **1** was used to treat thapsigargin-stimulated U2OS cells, which induced SGs through endoplasmic reticulum stress. The fluorescence signals of **1** were observed predominantly in the nucleus, with some new staining in the cytoplasm. In addition, TIA1 (as a SG marker) was found to co-localize with the cytoplasmic foci of **1** (Fig. 5). Moreover, after RNase treatment under similar conditions, no SG foci were observed (Fig. S7). This is a reasonable result, since SGs normally consist of RNA and SG-related proteins.

**Figure 6:**
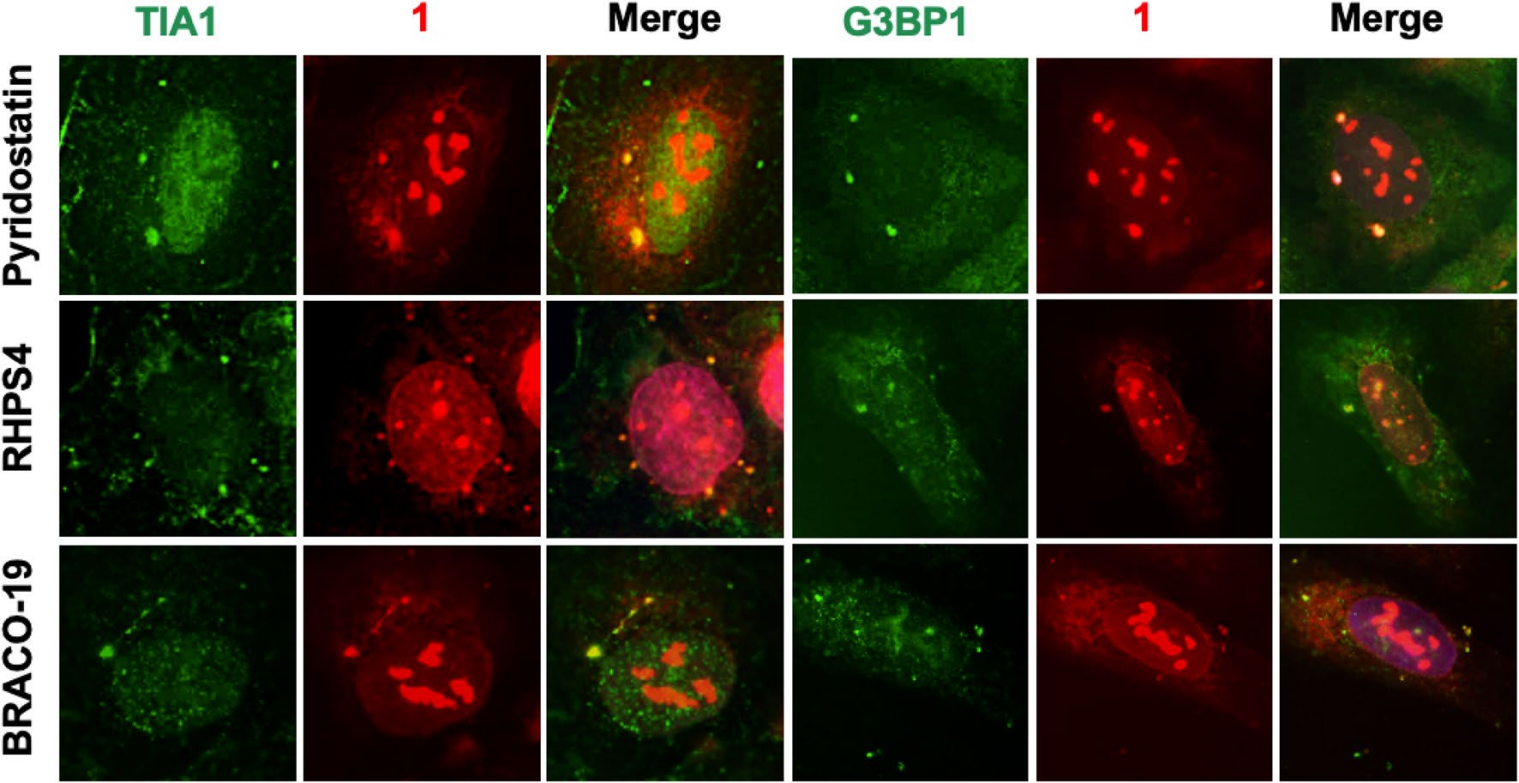
Co-localization of **1** with SG markers in G4-ligand-pretreated U2OS cells. Fluorescence was analyzed using the following detection channels: “TIA1 and G3BP1 channels” (ex. = 470/40 nm, em. = 525/50 nm) and “compound **1** channel” (ex. = 360/40 nm, em. = 605/70 nm).

Finally, we tested to induce SGs by treatment with G4 ligands and detect them using **1**. It was recently reported that a knockout of DHX36 (a type of G4 helicase) increases the amount of SGs^49^. In DHX36-depleted cells, G4 structures were probably stabilized because they were not unfolded by DHX36. Therefore, we attempted to stabilize G4s using known ligands. Three known G4 ligands, namely pyridostatin^19^, RHPS4^16^, and BRACO-19^15^, were pre-incubated with U2OS cells. In each case, the foci of **1** were co-localized with the SG markers, TIA1 and G3BP1 (Fig. 6). In the case of treatment with pyridostatin, SGs were also observed in HeLa cells (Fig. S8). Notably, pyridostatin-stimulated SGs were stained with DAPI and remained after pretreatment with RNase, unlike in the case of thapsigargin treatment (Fig. S9 and S10). These results indicated that pyridostatin-induced SGs probably contained DNA, which was consistent with the results obtained under oxidative stress conditions in a previous study^7^. According to these results, we have demonstrated that **1** is capable of detecting SGs and the G4-ligand-induced assembly of SGs containing DNA.

In conclusion, we designed and synthesized fused fluorophore **1**, which consists of BM and HBT, as a G4 ligand. Fluorophore **1** stabilized G4s strongly and selectively, and showed light-up properties; the fluorescence of **1** was quenched in water, whereas fluorescence emission was observed upon complexation between **1** and G4s. In cells, **1** was localized in the nucleoli, showing co-localization with the BG4 antibody. Moreover, we used **1** as a marker to detect G4s in SGs. Not only stimulation by reported condition, but also treatment with G4 ligands induced SGs, and we observed the SGs were co-localized with **1**. These results indicate that **1** is useful for detecting G4s in cells and can be utilized as an SG marker. Further investigations are currently underway for the elucidation of the molecular mechanisms of G4-ligand-induced SGs.

## Methods

### General

Analytical thin layer chromatography (TLC) was performed on glass plates coated with 0.25 mm 230–400 mesh silica gel containing a fluorescent indicator (Merck, #1.05715.0009). Silica gel column chromatography was performed using Kanto silica gel 60 (spherical, 40–100 µm). ^1^H and ^13^C NMR spectra were recorded on JEOL ECS-400 (400 MHz) and ECX-400 (400 MHz) spectrometers. For ^1^H NMR spectroscopy in CDCl_3_, the chemical shifts in the spectra were reported relative to tetramethylsilane (*δ* = 0). The other spectra were referenced internally according to the residual solvent signals of CDCl_3_ (^13^C NMR; *δ* = 77.0 ppm), dimethylsulfoxide-*d*_6_ (DMSO-*d*_6_) (^1^H NMR; *δ* = 2.49 ppm, ^13^C NMR; *δ* = 39.5 ppm). ^1^H NMR data were recorded as follows: chemical shift (*δ*, ppm), multiplicity (s, singlet; d, doublet; t, triplet; m, multiplet; br, broad), integration, coupling constant (Hz). ^13^C NMR data are reported in terms of the chemical shift (*δ*, ppm). Mass spectra were recorded with an Exactive (Thermo Fisher Scientific) spectrometer in electrospray ionization-mass spectrometry (ESI–MS) mode using methanol as the solvent. Starting materials, solvents, and reagents were obtained from commercial sources (Sigma Aldrich, TCI, Wako, Kanto Chemicals, and Nippoh Chemicals).

#### Synthesis of **2**

2-Iodoisophthalic acid (8.76 g, 30.0 mmol) and dimethylformamide (DMF) (one drop) were stirred at 50 °C in oxalyl chloride (15 mL) for 30 min. The solution was cooled, and then the oxalyl chloride was removed in vacuo. Then, the resulting product, 2-iodoisophthaloyl dichloride, and 2-amino-5-methoxybenzenethiol^50^ (10.2 g, 66.0 mmol) were stirred at 140 °C in *N*-methyl-2-pyrrolidone (NMP) (75 mL) for 1 h. After no further conversion was observed, water was added to the crude reaction mixture, which was then extracted with CHCl_3_. Then, the organic layer was washed with water and brine, dried over MgSO_4_, filtered, and concentrated in vacuo. The residue was purified using silica gel flash column chromatography with 0% to 5% AcOEt in toluene as the eluent to afford **2** (11.5 g, 72% yield) as a pale yellow solid; ^1^H NMR (400 MHz, CDCl_3_) *δ* = 8.03 (d, *J* = 9.0 Hz, 2H), 7.69 (d, *J* = 7.4 Hz, 2H), 7.55 (t, *J* = 7.4 Hz, 1H), 7.39 (d, *J* = 2.5 Hz, 2H), 7.15 (dd, *J* = 9.0, 2.5 Hz, 2H), 3.91 (s, 6H); ^13^C NMR (100 MHz, CDCl_3_) *δ* = 165.9, 158.0, 147.4, 140.8, 137.6, 132.0, 128.1, 124.3, 116.0, 103.7, 100.3, 55.8; HRMS (ESI) *m/z*: [M + H]^+^ Calcd for C_22_H_16_O_2_N_2_IS_2_ 530.9698; Found: 530.9692.

#### Synthesis of **3**

A solution of **2** (90.2 mg, 0.17 mmol) in CH_2_Cl_2_ (3 mL) was cooled at − 78 °C for 30 min and then BBr_3_ (3 mL) was added, and the resulting solution was stirred at room temperature for 3 h. After no further conversion was observed, MeOH and then Et_2_O were added to the crude reaction mixture, and the precipitated solid was filtered with Et_2_O. Then, the resulting residue, 60% NaH (34.0 mg, 0.85 mol), and *N*-Boc-2-chloroethylamine^51^ (152.7 mg, 0.85 mmol), which was prepared according to a reported procedure, were stirred at 80 °C in DMF (2 mL) for 17 h. After no further conversion was observed, 1 N aqueous HCl was added to the crude reaction mixture, which was then extracted with AcOEt. Then, the organic layer was washed with water and brine, dried over MgSO_4_, filtered, and concentrated in vacuo. The residue was purified using silica gel flash column chromatography with 0% to 100% AcOEt in hexane as the eluent to afford **3** (87.5 mg, 65% yield) as a pale yellow solid; ^1^H NMR (400 MHz, DMSO-*d*_6_) *δ* = 8.00 (d, *J* = 9.0 Hz, 2H), 7.80–7.76 (m, 2H), 7.76 (d, *J* = 2.5 Hz, 2H), 7.70–7.64 (m, 1H), 7.17 (d, *J* = 9.0, 2.5 Hz, 2H), 7.08 (t, *J* = 5.6 Hz, 2H), 4.07 (t, *J* = 5.6 Hz, 4H), 3.39–3.33 (m, 4H), 1.38 (s, 18H); ^13^C NMR (100 MHz, DMSO-*d*_6_) *δ* = 165.7, 156.8, 155.7, 147.0, 140.2, 137.3, 132.1, 128.6, 123.9, 116.5, 105.3, 101.1, 77.8, 67.1, 28.2; HRMS (ESI) *m/z*: [M + H]^+^ Calcd for C_34_H_38_O_6_N_4_IS_2_ 789.1288; Found: 789.1272.

#### Synthesis of **4**

According to a reported procedure^52^, **3** (157.8 mg, 0.2 mmol), Cu_2_O (14.3 mg, 0.1 mmol), TsOH·H_2_O (19.0 mg, 0.1 mmol), and Cs_2_CO_3_ (260.6 mg, 0.8 mmol) were stirred at 120 °C in H_2_O (3 mL) and DMSO (3 mL) for 14 h. After no further conversion was observed, 1 N aqueous HCl was added to the crude reaction mixture, which was then extracted with CHCl_3_. Then, the organic layer was washed with water and brine, dried over MgSO_4_, filtered, and concentrated in vacuo. The residue was purified using silica gel flash column chromatography with 0% to 10% AcOEt in toluene as the eluent to afford **4** (93.0 mg, 68% yield) as a yellow solid; ^1^H NMR (400 MHz, DMSO-*d*_6_) *δ* = 8.04 (d, *J* = 7.6 Hz, 2H), 7.89 (d, *J* = 8.8 Hz, 2H), 7.59 (d, *J* = 2.0 Hz, 2H), 7.12–7.00 (m, 5H), 4.0 (t, *J* = 5.4 Hz, 4H), 3.41–3.27 (m, 4H), 1.39 (s, 18H); ^13^C NMR (100 MHz, DMSO-*d*_6_) *δ* = 162.5, 156.6, 155.7, 154.4, 145.4, 135.4, 130.3, 122.7, 119.9, 119.1, 116.4, 104.9, 77.8, 67.0, 28.2; HRMS (ESI) *m/z*: [M + H]^+^ Calcd for C_34_H_39_O_7_N_4_S_2_ 679.2264; Found: 679.2255.

#### Synthesis of **5**

To a solution of **4** (223.3 mg, 0.33 mmol) in CH_2_Cl_2_ (50 mL) at 0 °C was added 1,3-diiodo-5,5-dimethylhydantoin (DIH) (125.0 mg, 0.33 mmol), and the resulting solution was stirred for 4 h. After no further conversion was observed, an aqueous Na_2_S_2_O_3_ solution was added to the crude reaction mixture, which was then extracted with CH_2_Cl_2_. Then, the organic layer was dried over Na_2_SO_4_, filtered, and concentrated in vacuo. The residue was purified using silica gel flash column chromatography with 0% to 10% AcOEt in toluene as the eluent to afford **5** (152.8 mg, 58% yield) as a yellow solid; ^1^H NMR (400 MHz, DMSO-*d*_6_) *δ* = 8.03–8.01 (m, 2H), 7.72 (d, *J* = 8.8 Hz, 2H), 7.48–7.46 (m, 2H), 7.08–7.05 (m, 2H), 6.95 (d, *J* = 8.3 Hz, 2H), 3.97–3.94 (m, 4H), 3.35–3.32 (m, 4H), 1.40 (s, 18H); ^13^C NMR (100 MHz, DMSO-*d*_6_) *δ* = 160.6, 156.6, 155.7, 153.9, 145.0, 137.0, 135.4, 122.7, 121.0, 116.4, 104.6, 81.9, 77.9, 67.0, 28.3; HRMS (ESI) *m/z*: [M − H]^−^ Calcd for C_34_H_36_O_7_N_4_IS_2_ 803.1112; Found: 803.1065.

#### Synthesis of **6**

According to a reported procedure^53^, **5** (95.0 mg, 118 µmol), Na_2_CO_3_ (37.5 mg, 354 µmol), *N*-formylsaccharin (74.8 mg, 354 µmol), triethylsilane (37.6 µL, 236 µmol), 1,4-bis(diphenylphosphino)butane (7.5 mg, 17.7 µmol), and Pd(OAc)_2_ (2.6 mg, 11.8 µmol) were stirred at 60 °C in DMF (5 mL) for 4 h. After no further conversion was observed, 1 N aqueous HCl was added to the crude reaction mixture, which was then extracted with CHCl_3_. Then, the organic layer was washed with water and brine, dried over MgSO_4_, filtered, and concentrated in vacuo. The residue was purified using silica gel flash column chromatography with 0% to 30% AcOEt in CH_2_Cl_2_ as the eluent to afford **6** (62.0 mg, 74% yield) as a yellow solid; ^1^H NMR (400 MHz, DMSO-*d*_6_) *δ* = 9.90 (s, 1H), 8.45 (s, 2H), 7.86 (d, *J* = 9.0 Hz, 2H), 7.59 (d, *J* = 2.2 Hz, 2H), 7.08 (t, *J* = 5.8 Hz, 2H), 7.05 (dd, *J* = 9.0, 2.2 Hz, 2H), 4.00 (t, *J* = 5.8 Hz, 4H), 3.34 (q, *J* = 5.8 Hz, 4H), 1.40 (s, 18H); ^13^C NMR (100 MHz, DMSO-*d*_6_) *δ* = 190.7, 161.6, 156.7, 155.8, 144.8, 135.4, 130.8, 122.7, 119.7, 116.7, 104.9, 77.9, 67.1, 28.3; HRMS (ESI) *m/z*: [M − H]^−^ Calcd for C_35_H_37_O_8_N_4_S_2_ 705.2079; Found: 705.2047.

#### Synthesis of **7**

A solution of **6** (62.0 mg, 87.7 µmol), 1,4-dimethylpyridinium iodide (30.9 mg, 131.6 µmol), and piperidine (10 µL) in EtOH (2 mL) was refluxed for 16 h. After no further conversion was observed, the solution was cooled and concentrated in vacuo. The residue was purified using silica gel flash column chromatography with 0% to 10% NH_3_ solution (3% NH_3_ in MeOH) in CHCl_3_ as the eluent to afford **7** (56.0 mg, 69% yield) as a red solid; ^1^H NMR (400 MHz, DMSO-*d*_6_) *δ* = 8.68 (s, 2H), 8.55 (d, *J* = 6.7 Hz, 2H), 8.15 (d, *J* = 15.7 Hz, 1H), 8.07 (d, *J* = 6.7 Hz, 2H), 7.81 (d, *J* = 9.0 Hz, 2H), 7.60 (d, *J* = 2.5 Hz, 2H), 7.15–7.01 (m, 5H), 4.09 (s, 3H), 4.06 (t, *J* = 5.6 Hz, 4H), 3.38–3.31 (m, 4H), 1.38 (s, 18H); ^13^C NMR (100 MHz, DMSO-*d*_6_) *δ* = 162.4, 155.7, 155.2, 153.8, 146.3, 143.7, 137.2, 129.5, 122.2, 121.6, 121.4, 115.3, 104.9, 77.8, 67.0, 46.0, 28.3; HRMS (ESI) *m/z*: [M + H]^+^ Calcd for C_42_H_46_O_7_N_5_S_2_ 796.2850; Found: 796.2833.

#### Synthesis of **1**

To a solution of **7** (56.0 mg, 60.6 µmol) in CH_2_Cl_2_ (1 mL) at room temperature was added trifluoroacetic acid (1 mL) and the resulting solution was stirred for 1 h. After no further conversion was observed, the solution was concentrated in vacuo and filtered with Et_2_O to afford **1** (59.0 mg, 99% yield) as a red solid; ^1^H NMR (400 MHz, DMSO-*d*_6_) *δ* = 8.72 (d, *J* = 6.5 Hz, 2H), 8.45 (s, 2H), 8.24 (s, 6H), 8.05 (d, *J* = 6.5 Hz, 2H), 8.01 (d, *J* = 15.9 Hz, 1H), 7.90 (d, *J* = 9.0 Hz, 2H), 7.68 (d, *J* = 2.2 Hz, 2H), 7.28 (d, *J* = 15.9 Hz, 1H), 7.12 (dd, *J* = 9.0, 2.2 Hz, 2H), 4.27 (t, *J* = 4.9 Hz, 4H), 4.19 (s, 3H), 3.38–3.21 (m, 4H); ^13^C NMR (100 MHz, DMSO-*d*_6_) *δ* = 162.5, 158.5, 158.2, 155.8, 152.6, 145.7, 144.5, 135.9, 129.6, 122.8, 122.6, 116.4, 105.3, 69.8, 65.0, 46.6, 38.4; HRMS (ESI) *m/z*: [M + H]^+^ Calcd for C_32_H_30_O_3_N_5_S_2_ 596.1780; Found: 596.1785.

### FRET melting assay

The dual-fluorescence-labeled oligonucleotides were purchased from Merck (Table S1). All nucleotides were dissolved in MilliQ water to prepare 100 μM stock solutions. Further dilutions of the oligonucleotides were performed using fluorescence resonance energy transfer (FRET) buffer (60 mM potassium cacodylate buffer (pH 7.4)), and dual-labeled DNA at 400 nM was annealed by heating at 96 °C for 2 min and then cooled to room temperature. A stock solution of **1** was prepared by dissolving it in DMSO (20 mM). It was further diluted to various concentrations (1.0–4.0 µM) with FRET buffer. The annealed DNA and the compound solutions (50 μL of each) were distributed in real-time polymerase chain reaction (PCR) tubes with 200 nM of labeled oligonucleotide for a total reaction volume of 100 μL. Measurements were carried out in triplicate with an excitation wavelength of 492 nm and a detection wavelength of 516 nm using an MX3005P Real-Time PCR system. Samples were kept at 25 °C for 5 min, and then the temperature was increased at 1 °C/min until reaching 95 °C. The emission of 6-carboxyfluorescein (FAM) was normalized between 0 and 1, and *T*_1/2_ was defined as the temperature at which the normalized emission was 0.5. The *T*_1/2_ was calculated from the average obtained from three experiments at each concentration of **1**.

### Absorption, excitation, and emission spectra

UV/vis absorption spectra were recorded on a Jasco V-730 UV/Vis spectrometer. The spectral band width was 1 nm. The fluorescence spectra were recorded on a Jasco FP-8300 spectrofluorometer. The slit widths of both monochromators were 1 nm. A stock solution of **1** was prepared by dissolving it in DMSO (20 mM). Further dilution was conducted using a suitable amount of water or various organic solvents. The absorption spectra of **1** were measured at 2 µM, and the fluorescence emission spectra of **1** were measured at 0.5 µM. All samples were measured at 25 °C.

### Fluorescence titrations

The fluorescence intensity was recorded with a Jasco FP-8300 spectrofluorometer using a quartz cell with an optical path length of 10 mm. A solution of **1** (50 nM) was prepared by diluting the stock solution (20 mM in DMSO) with 60 mM sodium cacodylate buffer containing 60 mM KCl (final volume of 2 mL). The freshly prepared solution of **1** was titrated with oligonucleotide solutions.

### Immunocytochemistry

H1299 (non-small cell lung cancer) cells were cultured in RPMI (Nacalai Tesque) and U2OS (osteosarcoma) cells were cultured in DMEM (Nacalai Tesque) supplemented with 10% fetal bovine serum (Gibco) and a 1% antibiotic–antimycotic-mixed solution (Nacalai Tesque) at 37 °C with 5% CO_2_. Cells were seeded on round glass coverslips and allowed to incubate for 72 h. Cells were washed with phosphate-buffered saline (PBS), fixed, and then permeabilized with 4% PFA and 0.2% Triton-X in PBS for 15 min at room temperature then cold MeOH for 10 min. Coverslips were blocked with Blocking One Histo (Nacalai Tesque) and incubated with the primary antibody for 2 h at room temperature. In the case of BG4, coverslips were subsequently incubated with anti-FLAG antibody for 2 h at room temperature. After incubation with the secondary antibody and 4′,6-diamidino-2-phenylindole (DAPI) (1 ng/mL) for 1 h at room temperature, the coverslips were treated with **1** (2 µM) for 1 h at room temperature. For enzymatic treatments, coverslips were incubated after permeabilization with 0.12 U/µL DNase I (Nippon Gene) or with 100 mg/mL RNase A (Nippon Gene) for 1 h at 37 °C. For thapsigargin treatment, cells were incubated at 1 µM for 1 h before fixation. For G4 ligand treatments, cells were incubated with the ligands (pyridostatin (5 µM), RHPS4 (10 µM), or BRACO (10 µM)) for 24 h before fixation. The cells were mounted with fluorescence mounting medium (Agilent), and digital images were recorded with BZ-X710 (Keyence). Fluorescence emission was measured on the following detection channels (Keyence): OP-87762 (ex. = 360/40 nm, em. = 460/50 nm), OP-87763 (ex. = 470/40 nm, em. = 525/50 nm), or OP-87765 (ex. = 560/40, em. = 630/75 nm). For the fluorescence detection of **1**, we used a custom channel consisting of a 360/40 nm filter for excitation and a 605/70 nm filter for emission. The following primary and secondary antibodies were used in this study: anti-G4 BG4 (1:50 dilution, MABE917, Merck), anti-FLAG (1:1000 dilution, F1804, Merck), anti-fibrillarin (1:400 dilution, 2639, Cell Signaling Technology), anti-G3BP (1:200 dilution, 611,126, BD Transduction Laboratories), anti-TIA1 (1:100 dilution, SC-166247, Santa Cruz Biotechnology), anti-mouse Alexa 488-conjugated (1:500–1000 dilution, A11029, Thermo Fisher Scientific), and anti-rabbit Alexa 594-conjugated (1:500–1000 dilution, A11037, Thermo Fisher Scientific).

## Supplementary Information

Supplementary Information.

## Data Availability

All data generated or analyzed during this study are included in this published article [and its supplementary information files].
