# Supplementary material for: Development of a novel light-up probe for detection of G-quadruplexes in stress granules
Source: Sci Rep. 2022 Jul 28;12:12892. doi: 10.1038/s41598-022-17230-y (PMC9334577; doi:10.1038/s41598-022-17230-y)
Supplement: Supplementary file 1 — Supplementary Information. [file 41598_2022_17230_MOESM1_ESM.docx]

**Development of a Novel Light-up Probe for Detection of**

**G-quadruplexes in Stress Granules**

Keisuke Iida,* Natsumi Suzuki, Ayano Sasaki, Shunsuke Ishida, Takayoshi Arai

Soft Molecular Activation Research Center (SMARC)

Chiba Iodine Resource Innovation Center (CIRIC)

Molecular Chirality Research Center (MCRC)

and Department of Chemistry, Graduate School of Science, Chiba University,

1-33 Yayoi, Inage, Chiba 263-8522, Japan.

Supplementary information

Contents

1. ^1^H and ^13^C NMR spectra S3
2. FRET melting assay S17
3. Absorption, excitation, and emission spectra of **1** S18
4. Fluorescence titrations S21
5. Immunocytochemistry S22
6. **^1^H and ^13^C NMR spectra**

**^1^H NMR (400 MHz, CDCl_3_)**

**^13^C NMR (100 MHz, CDCl_3_)**

**^1^H NMR (400 MHz, DMSO-*d*_6_)**

**^13^C NMR (100 MHz, DMSO-*d*_6_)**

**^1^H NMR (400 MHz, DMSO-*d*_6_)**

**^13^C NMR (100 MHz, DMSO-*d*_6_)**

**^1^H NMR (400 MHz, DMSO-*d*_6_)**

**^13^C NMR (100 MHz, DMSO-*d*_6_)**

**^1^H NMR (400 MHz, DMSO-*d*_6_)**

**^13^C NMR (100 MHz, DMSO-*d*_6_)**

**^1^H NMR (400 MHz, DMSO-*d*_6_)**

**^13^C NMR (100 MHz, DMSO-*d*_6_)**

**^1^H NMR (400 MHz, DMSO-*d*_6_)**

**^13^C NMR (100 MHz, DMSO-*d*_6_)**

1. **FRET melting assay**

**Table S1.** Oligonucleotides used in FRET melting assay

| Nature | Name | Sequence | Length | Topology |
| --- | --- | --- | --- | --- |
| RNA | F-VEGF-T | FAM-r[^5'^GGAGGAGGGGAGGAGGA^3'^]-TAMRA | 17 | Parallel^[1]^ |
|  | F-TRF2-T | FAM-r[^5'^CGGGAGGGCGGGGAGGGC^3'^]-TAMRA | 18 | Parallel^[2]^ |
|  | F-TERRA-T | FAM-r[^5'^GGGUUAGGGUUAGGGUUAGGG^3'^]-TAMRA | 21 | Parallel^[3]^ |
| DNA | F-telo-T | FAM-d[^5'^GGGTTAGGGTTAGGGTTAGGG^3'^]-TAMRA | 21 | Hybrid^[4]^ |
|  | F-myc-T | FAM-d[^5'^GAGGGTGGGGAGGGTGGGGAAG^3'^]-TAMRA | 22 | Parallel^[5]^ |
|  | F-kit-T | FAM-d[^5'^GGGAGGGCGCTGGGAGGAGGG^3'^]-TAMRA | 21 | Parallel^[6]^ |
|  | F-thr-T | FAM-d[^5'^GGTTGGTGTGGTTGG^3'^]-TAMRA | 15 | Anti-parallel^[7]^ |
|  | F-ds26-T | FAM-d[^5'^TATAGCTATATTTTTTTATAGCTATA^3'^]-TAMRA | 26 | Duplex |

**Table S2.** Summary of results in FRET melting assay

| Nature | Name | *T*_1/2_ (Δ*T*_1/2_), (℃) | | | | |
| --- | --- | --- | --- | --- | --- | --- |
|  |  | 0 µM | 0.5 µM | 1 µM | 1.5 µM | 2.0 µM |
| RNA | VEGF | 63.4±0.1 | 69.6±1.7  (6.2±1.7) | 75.8±0.1  (12.4±0.2) | 79.9±0.4  (16.5±0.2) | 82.7±0.2  (19.3±0.2) |
|  | TRF2 | 73.5±0.6 | 85.0±0.1  (11.5±0.8) | 89.4±0.1  (15.9±0.7) | 90.8±0.0  (17.3±0.6) | 91.1±0.1  (17.6±0.5) |
|  | TERRA | 54.8±0.3 | 70.4±0.2  (15.5±0.1) | 80.5±0.8  (25.7±0.1) | 84.7±0.1  (29.9±0.1) | 88.9±0.4  (34.1±0.1) |
| DNA | telo | 58.8±0.9 | 71.1±0.7  (12.3±0.4) | 78.3±0.3  (19.5±0.8) | 84.0±0.2  (25.2±0.8) | 87.8±0.4  (29.0±0.7) |
|  | myc | 48.3±0.2 | 65.4±0.3  (17.1±0.6) | 71.6±2.0  (23.1±1.7) | 75.3±0.1  (26.9±0.2) | 79.8±0.4  (31.4±0.2) |
|  | kit | 62.6±1.7 | 77.1±0.5  (14.6±1.3) | 82.4±0.2  (19.8±1.6) | 85.2±0.3  (22.6±1.4) | 87.0±0.1  (24.4±1.6) |
|  | thr | 51.2±0.6 | 53.9±0.9  (2.7±0.3) | 57.3±0.3  (6.1±0.3) | 61.6±1.2  (10.4±0.6) | 64.2±0.3  (13.0±0.7) |
|  | ds26 | 58.1±0.8 | 59.1±0.3  (1.0±1.1) | 59.8±0.2  (1.7±1.0) | 60.3±0.1  (2.2±0.9) | 61.4±0.6  (3.3±1.3) |

**Figure S1.** *T*_1/2_ values of oligonucleotides (0.2 μM) were determined using FRET melting assay in the absence or presence of **1** (0.5–2.0 μM) in 60 mM potassium cacodylate buffer (pH 7.4).

1. **Absorption, excitation, and emission spectra of 1**

**Figure S2.** (A) Absorbance and (B) fluorescence emission spectra of **1** in various solvents. Correlation between (C) absorption and (D) fluorescence emission wavelength of peaks and E_T_^N^ (normalized solvent polarity parameter).^[12]^

**Figure S3.** Excitation and emission spectra of **1** in THF/water solutions at increasing THF volume fractions. Dotted lines show excitation spectra and solid lines show emission spectra.

**Figure S4.** Results of UV-vis titration of **1** (2 µM) in water upon addition of sodium dodecyl sulfate.

As shown in Figure S2, two absorption bands appeared for some solvents. A previous study reported that HBT derivatives give two absorption bands, which were ascribed to the keto (longer wavelength) and enol (shorter wavelength) forms.^[11,12]^ Moreover, protic solvents form hydrogen bonds with HBT, which disturbs the intramolecular hydrogen bonds and inhibits ESIPT. Therefore, the fluorescence emission of the enol form is predominant in water (Figure S3). On the other hand, new absorption bands (corresponding to the keto form) appeared at 575 nm when mixed with THF in increasing proportions to water (Figure S3). Their behavior is similar to that seen in the results of the G4 titration of **1**, and is probably due to the recovery of ESIPT. Additionally, the fluorescence intensity of the enol form was much weaker than that of the keto form. There were two possible reasons for this: (i) the nature of the 2,6-dibenzothiazolylphenol skeleton^[11,12]^ and (ii) aggregation-caused quenching (ACQ). To investigate ACQ, sodium dodecyl sulfate (SDS) was added to an aqueous solution of **1**. The titration results showed that the surfactants disassembled aggregated **1**, based on the decrease in the intensity of the bands near 440 nm (Figure S4). Based on these results, we concluded that the light-up properties of **1** are due to ACQ and on/off switching of ESIPT through binding to G4s.

1. **Fluorescence titrations**

**Table S3.** Oligonucleotides used in fluorescence titrations

| Nature | Name | Sequence | Length | Topology |
| --- | --- | --- | --- | --- |
| RNA | VEGF | r[^5'^GGAGGAGGGGAGGAGGA^3'^] | 17 | Parallel^[1]^ |
|  | TRF2 | r[^5'^CGGGAGGGCGGGGAGGGC^3'^] | 18 | Parallel^[2]^ |
|  | TERRA | r[^5'^GGGUUAGGGUUAGGGUUAGGG^3'^] | 21 | Parallel^[3]^ |
| DNA | telo | d[^5'^GGGTTAGGGTTAGGGTTAGGG^3'^] | 21 | Hybrid^[4]^ |
|  | myc | d[^5'^GAGGGTGGGGAGGGTGGGGAAG^3'^] | 22 | Parallel^[5]^ |
|  | kit | d[^5'^GGGAGGGCGCTGGGAGGAGGG^3'^] | 21 | Parallel^[6]^ |
|  | thr | d[^5'^GGTTGGTGTGGTTGG^3'^] | 15 | Anti-parallel^[7]^ |
|  | Bom17 | d[^5'^GGTTAGGTTAGGTTAGG^3'^] | 17 | Anti-parallel^[8,9]^ |
|  | PARP1 | d[^5'^TGGGGGCCGAGGCGGGGCTTGGG^3'^] | 23 | Hybrid^[10]^ |
|  | ds26 | d[^5'^TATAGCTATATTTTTTTATAGCTATA^3'^] | 26 | Duplex |

References

[1] M. J. Morris, Y. Negishi, C. Pazsint, J. D. Schonhoft, S. Basu, *J. Am. Chem. Soc.* **2010**, *132*, 17831–17839; [2] D. Gomez, A. Guedin, J. L. Mergny, B. Salles, J. F. Riou, M. P. Teulade-Fichou, P. Calsou, *Nucleic Acids Res.* **2010**, *38*, 7187–7198; [3] H. Martadinata, A. T. Phan, *Biochemistry* **2013**, *52*, 2176–2183; [4] A. T. Phan, V. Kuryavyi, K. N. Luu, D. J. Patel, *Nucleic Acids Res.* **2007**, *35*, 6517–6525; [5] A. Ambrus, D. Chen, J. Dai, R. A. Jones, D. Yang, *Biochemistry* **2005**, *44*, 2048–2058; [6] A. T. Phan, V. Kuryavyi, S. Burge, S. Neidle, D. J. Patel, *J. Am. Chem. Soc.* **2007**, *129*, 4386–4392; [7] V. M. Marathias, K. Y. Wang, S. Kumar, T. Q. Pham, S. Swaminathan, P. H. Bolton, *J. Mol. Biol.* **1996**, *260*, 378–394; [8] S. Amrane, R. W. Ang, Z. M. Tan, C. Li, J. K. Lim, J. M. Lim, K. W. Lim, A. T. Phan, *Nucleic Acids Res.* **2009**, *37*, 931–938; [9] A. D. Rache, J.-L. Mergny, *Biochimie*, **2015**, *115*, 195–202, [10] A. Sengar, J. J. Vandana, V. S. Chambers, M. Di Antonio, F. R. Winnerdy, S. Balasubramanian, A. T. Phan, *Nucleic Acids Res.* **2019**, *47*, 1564–1572; [11] C. Reichardt, *Chem. Rev.* **1994**, *94*, 2319–2358; [12] K. Sakai, T. Ishikawa, T. Akutagawa, *J. Mater. Chem. C* **2013**, *1*, 7866–7871.

1. **Immunocytochemistry**


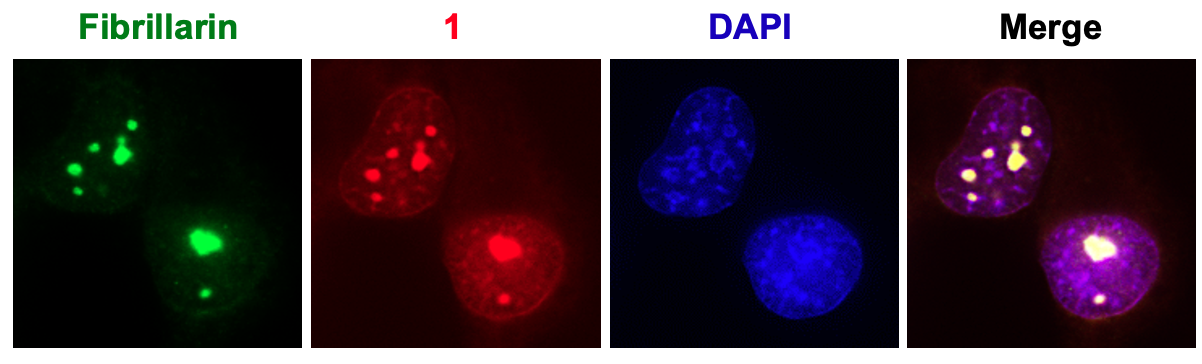


**Figure S5.** Co-localization of **1** and anti-fibrillarin antibody in H1299 cells. Fluorescence was analyzed using the following detection channels: "compound 1 channel" (ex. = 360/40 nm, em. = 605/70 nm), "Fibrillarin channel" (ex. = 560/40 nm, em. = 630/75 nm), and "DAPI channel" (ex. = 360/40 nm, em. = 460/50 nm).


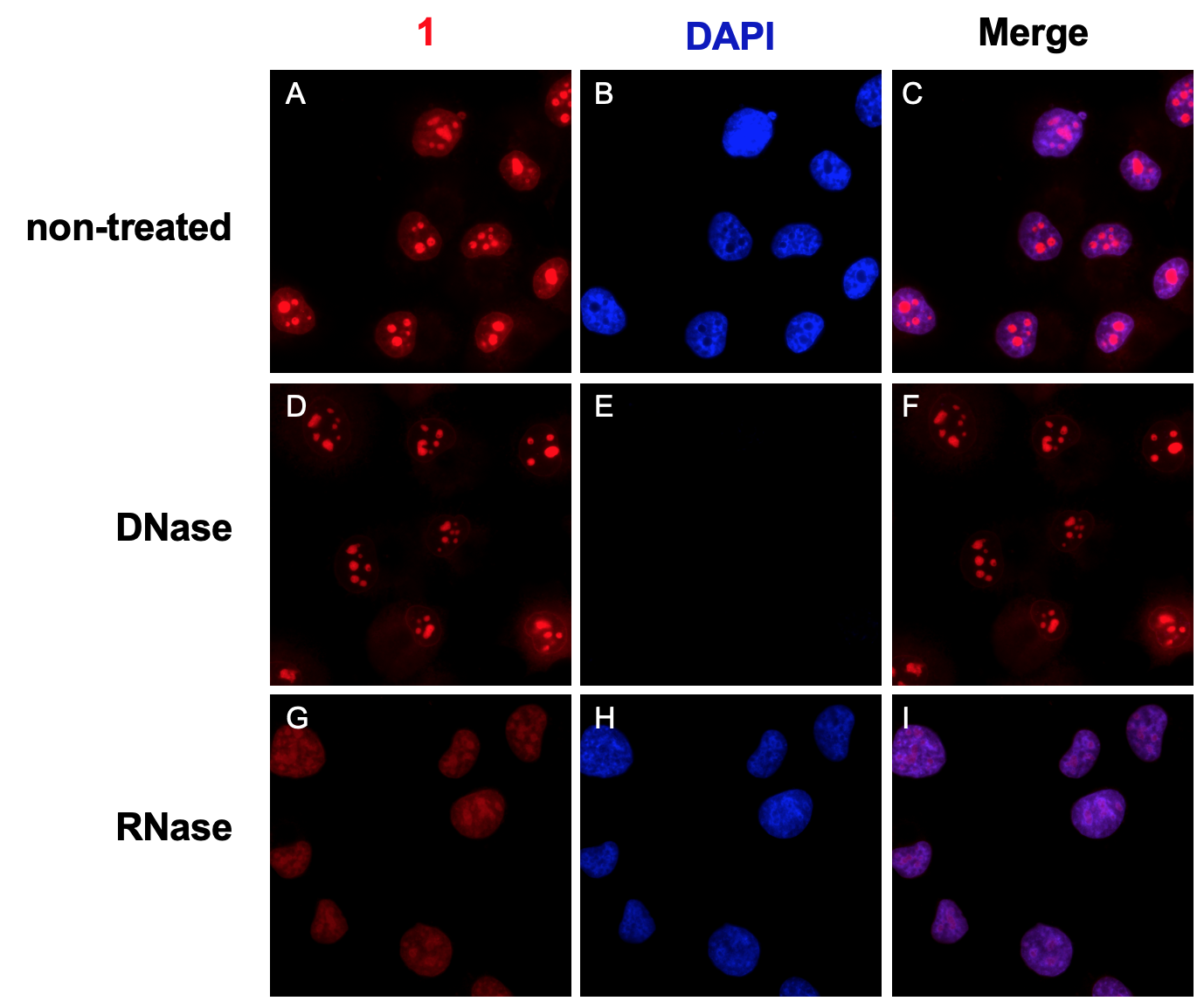


**Figure S6.** Control experiments to confirm the binding of **1** to nucleic acids in H1299 cells. Fluorescence was analyzed using the following detection channels: "compound 1 channel" (ex. = 360/40 nm, em. = 605/70 nm) and "DAPI channel" (ex. = 360/40 nm, em. = 460/50 nm).


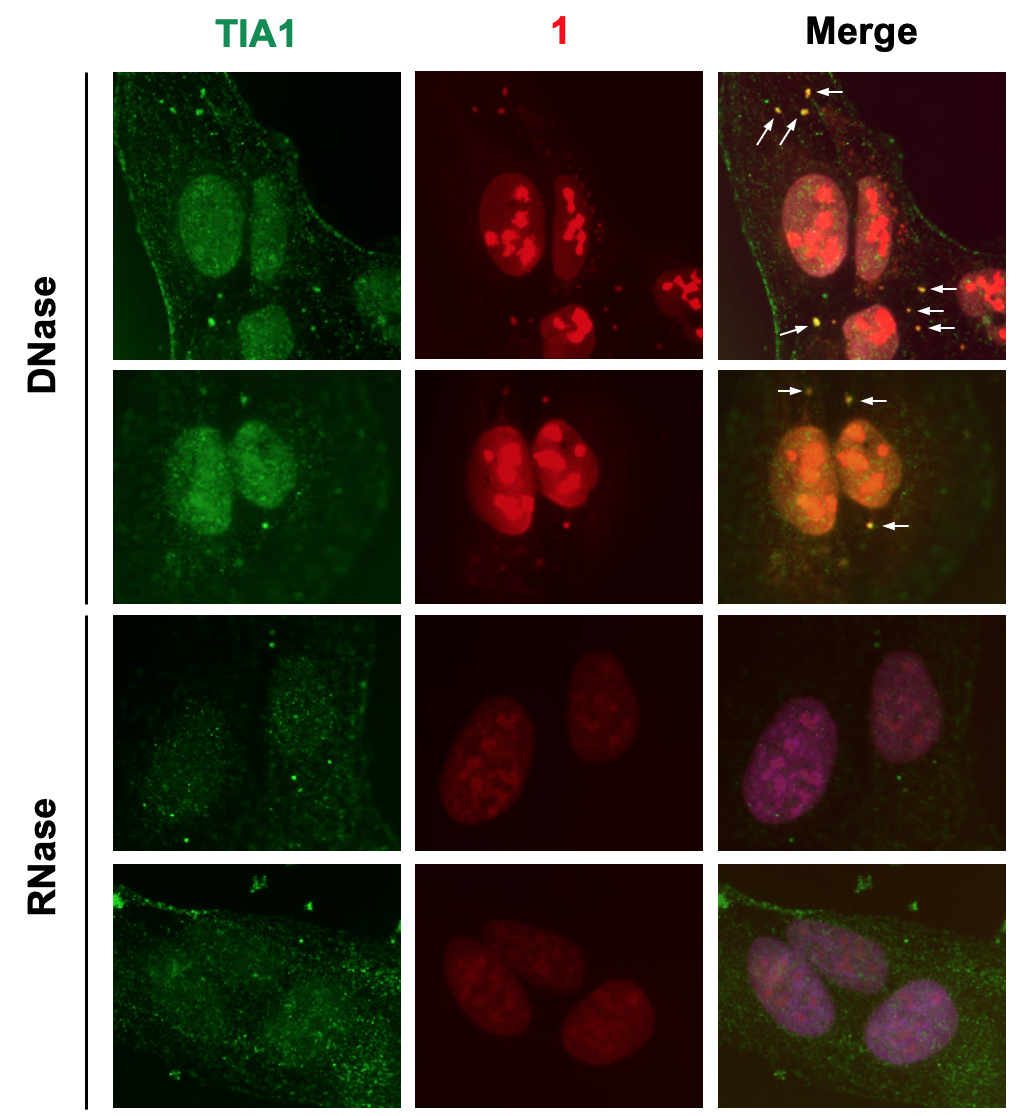


**Figure S7.** Control experiments to confirm binding of **1** to nucleic acids in stress granules of thapsigargin-pretreated U2OS cells. Fluorescence was analyzed using the following detection channels: "TIA1 channel" (ex. = 470/40 nm, em. = 525/50 nm), "compound 1 channel" (ex. = 360/40 nm, em. = 605/70 nm), and "DAPI channel" (ex. = 360/40 nm, em. = 460/50 nm).


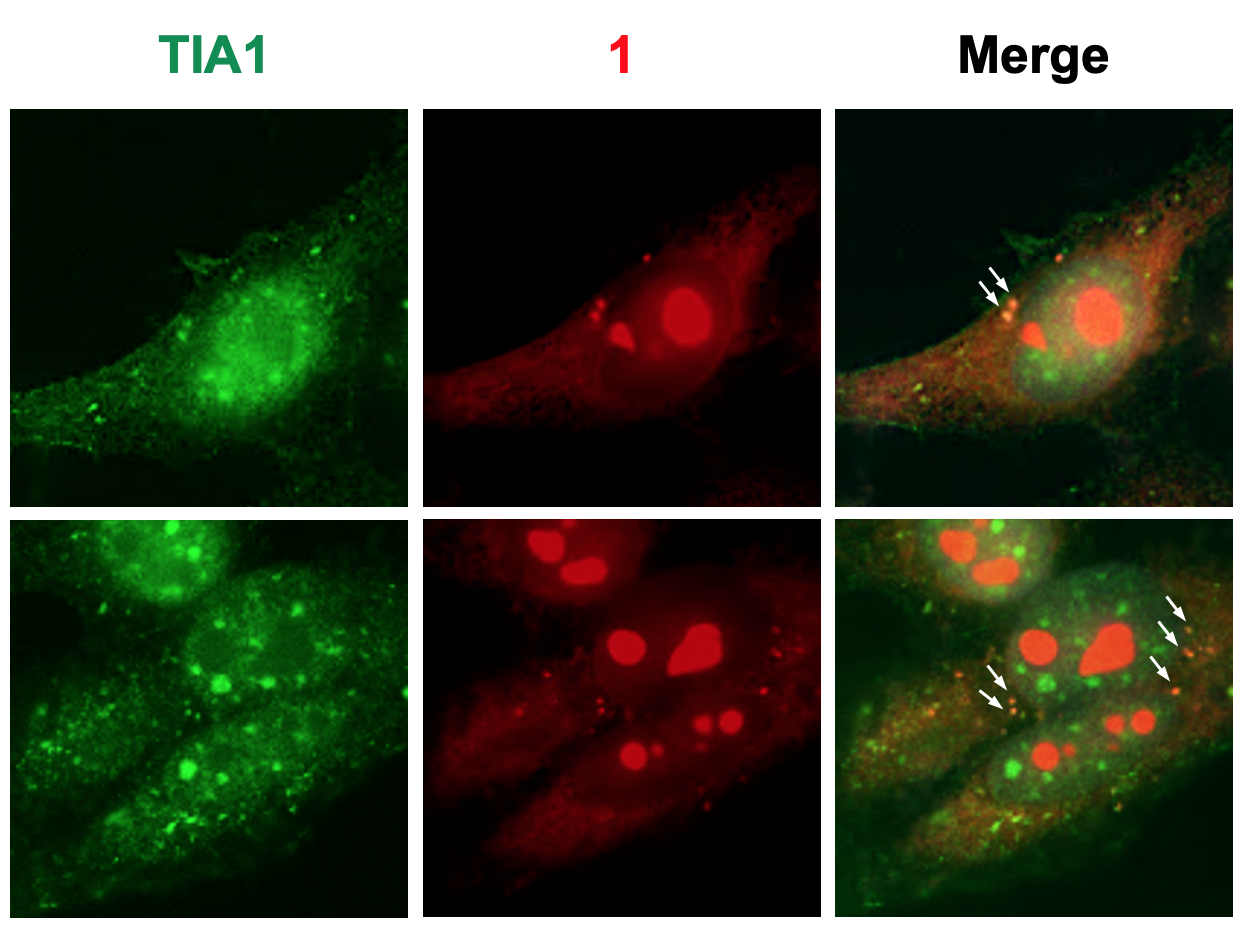


**Figure S8.** Co-localization of **1** with TIA1 in pyridostatin-pretreated HeLa cells. Fluorescence was analyzed using the following detection channels: "TIA1 channel" (ex. = 470/40 nm, em. = 525/50 nm) and "compound 1 channel" (ex. = 360/40 nm, em. = 605/70 nm).


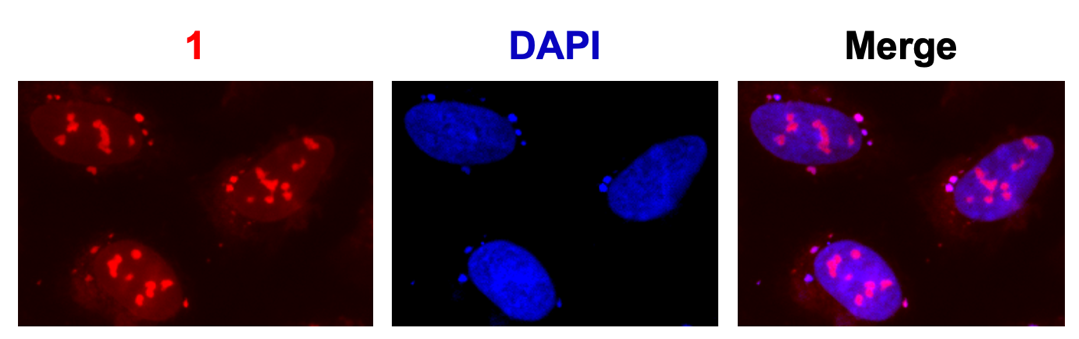


**Figure S9.** Co-localization of **1** with DAPI in pyridostatin-pretreated U2OS cells. Fluorescence was analyzed using the following detection channels: "compound 1 channel" (ex. = 360/40 nm, em. = 605/70 nm), and "DAPI channel" (ex. = 360/40 nm, em. = 460/50 nm).


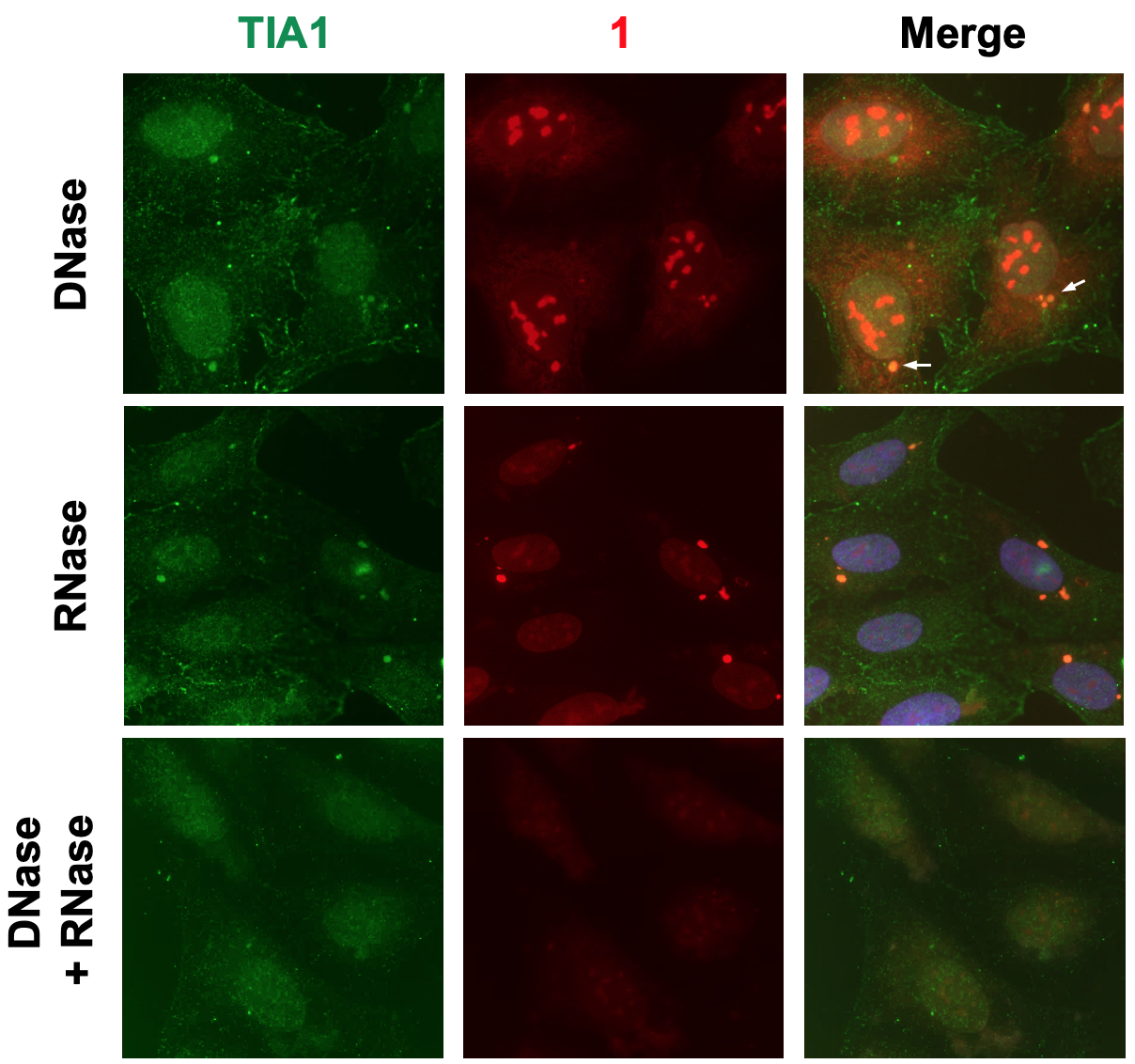


**Figure S10.** Control experiments to confirm binding of **1** to nucleic acids in stress granules of pyridostatin-pretreated U2OS cells. Fluorescence was analyzed using the following detection channels: "TIA1 channel" (ex. = 470/40 nm, em. = 525/50 nm) and "compound 1 channel" (ex. = 360/40 nm, em. = 605/70 nm).
